# Norcoclaurine Synthase: Mechanism of an Enantioselective Pictet-Spengler Catalyzing Enzyme

**DOI:** 10.3390/molecules15042070

**Published:** 2010-03-24

**Authors:** Alessandra Bonamore, Marco Barba, Bruno Botta, Alberto Boffi, Alberto Macone

**Affiliations:** 1 Dipartimento di Scienze Biochimiche, Università “Sapienza” P. Aldo Moro 5, 00185, Rome, Italy; 2 Dipartimento di Studi di Chimica e Tecnologia delle Sostanze Biologicamente Attive, Università “Sapienza” P. Aldo Moro 5, 00185, Rome, Italy

**Keywords:** norcoclaurine synthase, (S)-norcoclaurine, Pictet-Spengler condensation, bifunctional catalysis, green synthetic process

## Abstract

The use of bifunctional catalysts in organic synthesis finds inspiration in the selectivity of enzymatic catalysis which arises from the specific interactions between basic and acidic amino acid residues and the substrate itself in order to stabilize developing charges in the transition state. Many enzymes act as bifunctional catalysts using amino acid residues at the active site as Lewis acids and Lewis bases to modify the substrate as required for the given transformation. They bear a clear advantage over non-biological methods for their ability to tackle problems related to the synthesis of enantiopure compounds as chiral building blocks for drugs and agrochemicals. Moreover, enzymatic synthesis may offer the advantage of a clean and green synthetic process in the absence of organic solvents and metal catalysts. In this work the reaction mechanism of norcoclaurine synthase is described. This enzyme catalyzes the Pictet-Spengler condensation of dopamine with 4-hydroxyphenylacetaldehyde (4-HPAA) to yield the benzylisoquinoline alkaloids central precursor, (S)-norcoclaurine. Kinetic and crystallographic data suggest that the reaction mechanism occurs according to a typical bifunctional catalytic process.

## 1. Enzymatic Bifunctional Catalysis

Acid-base bifunctional catalysis is considered an efficient and reliable strategy in small molecule asymmetric catalysis. Bifunctional catalysts are able to simultaneously bind and activate two reacting substrates, allowing the development of stereoselective strategies for the synthesis of chiral compounds [1]. 

From the viewpoint of reaction mechanism, the interaction of acidic and basic moieties with the substrate can be simultaneous or subsequent. The simultaneous one can be further divided into “concerted” and the “go-together” mechanisms. In the concerted mechanism, the acid-base interaction takes place simultaneously in different positions of the substrate, whereas the go-together strategy implies that the first substrate activated by the acidic site reacts with the second substrate activated by the basic site [2].

The use of bifunctional catalysts in organic synthesis finds inspiration in the selectivity of enzymatic catalysis which arises from the specific interactions between basic and acidic aminoacid residues and the substrate itself in order to stabilize developing charges in the transition state [1]. Enzymes, in fact, are known to be chemoselective, regioselective and enantioselective and these features renders them ideal catalysts in chiral synthesis. For this reason many organic chemists have recently focused their attention in the use of enzymes as bifunctional catalysts, now widely recognized as practical alternatives to traditional (non-biological) organic synthesis [3].

Many enzymes act as bifunctional catalysts using aminoacidic residues at the active site as Lewis acids and Lewis bases to modify the substrate as required for the given transformation. The action of the enzyme can be exerted by activating nucleophile and electrophile groups, or by stabilizing leaving groups. Histidine is often the residue involved in these acid/base reactions, since it has a pKa close to neutral pH and can therefore both accept and donate protons. Anyway, more than one proton exchanger can be involved in such reaction, as widely demonstrated by site directed mutagenesis studies [4,5]. 

Bifunctional biocatalysts including hydrolases, racemases, lipases, isomerases, *etc*., are currently used in industrial biotransformation. Mandelate racemase, for example, is a useful catalyst for the racemization of stereochemically stable α-hydroxycarboxylic acids, such as mandelic acid and its derivatives, at neutral pH and ambient temperature [6]. Hydantoin racemase is the key enzyme for the production of optically pure D- and L-amino acids, valuable intermediates for the synthesis of antibiotics, sweeteners, pesticides, pharmaceuticals and biologically active peptides [7]. In some cases racemase catalyzed reaction has been coupled with a lipase to furnish a two-enzyme deracemization process [8]. Lipases, whose role in nature is to catalyse the hydrolysis of triacyl glycerides, have been also successfully used as transesterification catalysts for stereoselective acylation and kinetic resolution of alcohols [9]. All these enzymes represent a new class of chiral catalysts useful for a broad range of selective organic transformations. They bear a clear advantage over non-biological methods for their ability to tackle problems related to the synthesis of enantiopure compounds as chiral building blocks for drugs and agrochemicals. To this purpose screening for novel enzymes capable of catalyzing new enantioselective reactions is constantly needed. In addition, the discovery of new enzymes will provide clues for designing new green chemistry processes. 

## 2. Benzylisoquinoline Alkaloids Production

Benzylisoquinoline alkaloids are a complex and diverse group of natural products consisting of more than 2,500 known structures, found in distantly related families including the Papaveraceae, Ranunculaceae, Berberidaceae, Fumariaceae, and Menispermaceae [10]. These compounds, naturally involved in the chemical defense of plants against herbivores and pathogens, are also pharmacologically active. Morphine, which is the most important member of the group of benzylisoquinoline alkaloids, is a natural product with high medicinal significance. Like morphine, codeine is used as an analgesic. Berberine and sanguinarine, instead, are used as antimicrobials; papaverine and (+)-tubocurarine work as muscle relaxants. The structural complexity of these pharmaceuticals often renders chemical synthesis impractical as an alternative to extraction of the secondary metabolite from cultivated plants for their commercial production. 

## 3. Bifunctional Catalysis in the Enzymatic Synthesis of (*S*)-Norcoclaurine

**Scheme 1:**
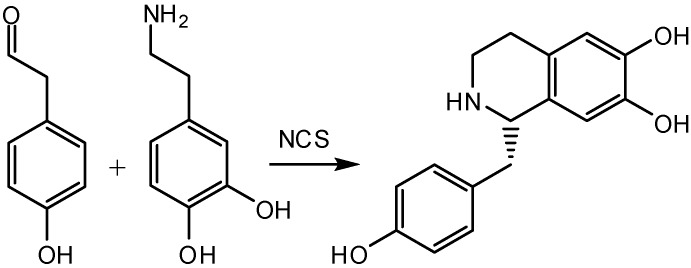
(S)-norcoclaurine biosynthesis.

The enzymatic pathways leading to the amazing diversity of benzylisoquinoline derivatives have been shown to originate from a common route in which the first committed step consists of the Pictet-Spengler condensation of dopamine with 4-hydroxyphenylacetaldehyde (4-HPAA) to yield the benzylisoquinoline central precursor, (*S*)-norcoclaurine (CAS 5843-65-2) (Scheme 1) [11,12]. 

The enzyme catalyzing this reaction has been identified as norcolaurine synthase (NCS, EC 4.2.1.78) and its crystallographic structure has been recently solved [13,14]. The crystallographic data, obtained in complex with dopamine, the natural substrate, and p-hydroxybenzaldehyde, a non reactive substrate analogue, provided a snapshot of the initial step of the reaction mechanism, offering a novel powerful example of enzymatic bifunctional catalysis. The reaction is highly stereospecific, and the chirality of the (*S*)-norcoclaurine product is essential to drive the intricate pathway of substrate stereoselective enzymatic reactions toward the terminal metabolites.

**Figure 1:**
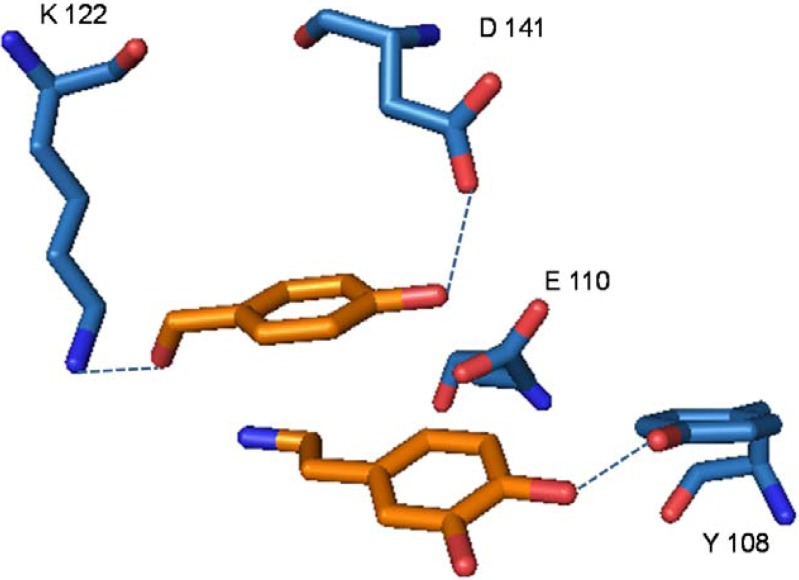
Amino acid residues involved in NCS catalysis.

Analysis of the X-ray structures NCS and NCS complex with the substrate analogue p-hydroxy benzaldehyde (PHB) and dopamine revealed a tetrameric assembly, in which the overall fold adopted by each monomer is analogue to proteins belonging to the Bet v1-like superfamily [15]. The characterizing secondary elements consist of a seven-stranded antiparallel β-sheets wrapped around a long C-terminal helix (α3) and two smaller α-helical segments (α1 and α2) [15]. Each monomer shows an accessible cleft, formed by a series of hydrophobic residues and a polar patch located at the entrance of the cavity. X-ray data obtained on crystals soaked with the dopamine substrate and PHB indicate that the two molecules adopt a stacked configuration with the respective aromatic rings lying on almost parallel planes. PHB carbonyl oxygen forms an hydrogen bond with Lys^122^ amino group, whereas the phenolic oxygen is in contact with the carboxyl moiety of Asp^141^. Dopamine is hold in place by the stacking interaction with PHB and by hydrogen bonding of the C-1 phenol hydroxyl with the Tyr^108^ phenol hydroxyl. Most significantly, dopamine C-5 carbon atom lies close to the carboxyl group of Glu^110^, suggesting a key role for this residue in the catalytic mechanism (Figure 1) [14].

The geometry of the NCS active site is indeed dominated by the presence of three strong proton exchangers, Lys^122^, Asp^141^, and Glu^110^, and of a hydrogen bonding donor, Tyr^108^. These residues shape the binding site of the two aromatic substrates and suggest the typical acid-base mechanism (see Figure 1), that matches closely the classical two-step Pictet-Spengler scheme and leads to the stereospecific ring closure to yield (*S*)-norcoclaurine [14]. 

**Scheme 2:**
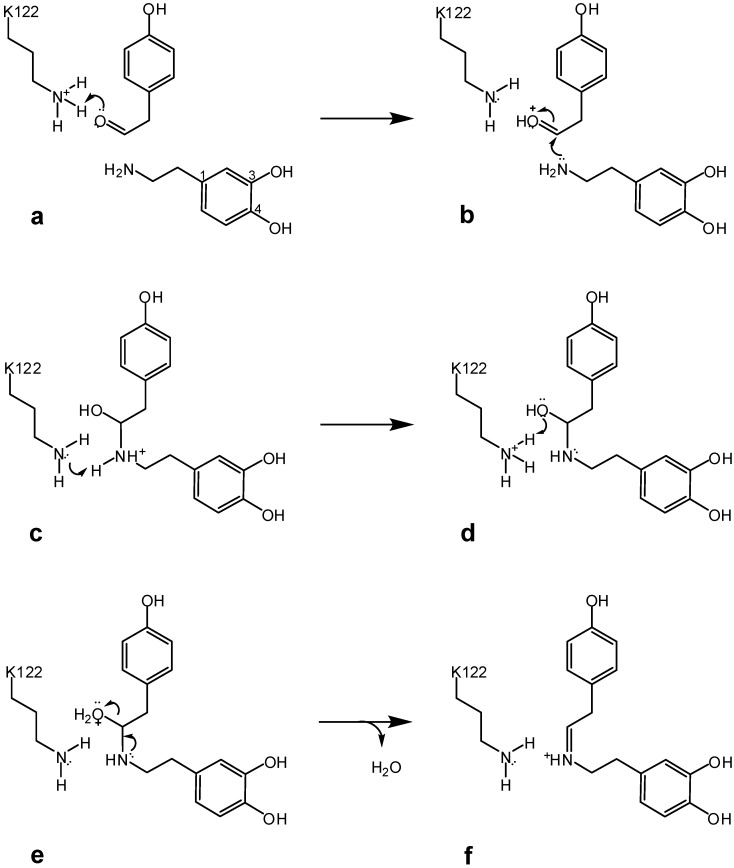
Mechanism of NCS catalyzed iminium ion formation as intermediate in (S)-norcoclaurine synthesis.

The first aminoacid involved in the formation of (*S*)-norcoclaurine is Lys^122^. This residue performs a tandem acid-base catalysis being able to donate and abstract a proton to/from the substrates (Scheme 2). According to this mechanism, the interaction between the amino group of Lys^122^ and the carbonyl oxygen of the aldehyde allows a proton transfer from the ammonium ion to the carbonyl oxygen and consequent nucleophilic attack of dopamine (steps a and b). Afterwards, lysine abstracts a second proton from the reaction intermediate leading to the formation of the carbinolamine moiety (step c). Lys^122^ is also involved in the water molecule release from the carbinolamine, with the consequent formation of the iminium ion (steps d to f). 

**Scheme 3:**
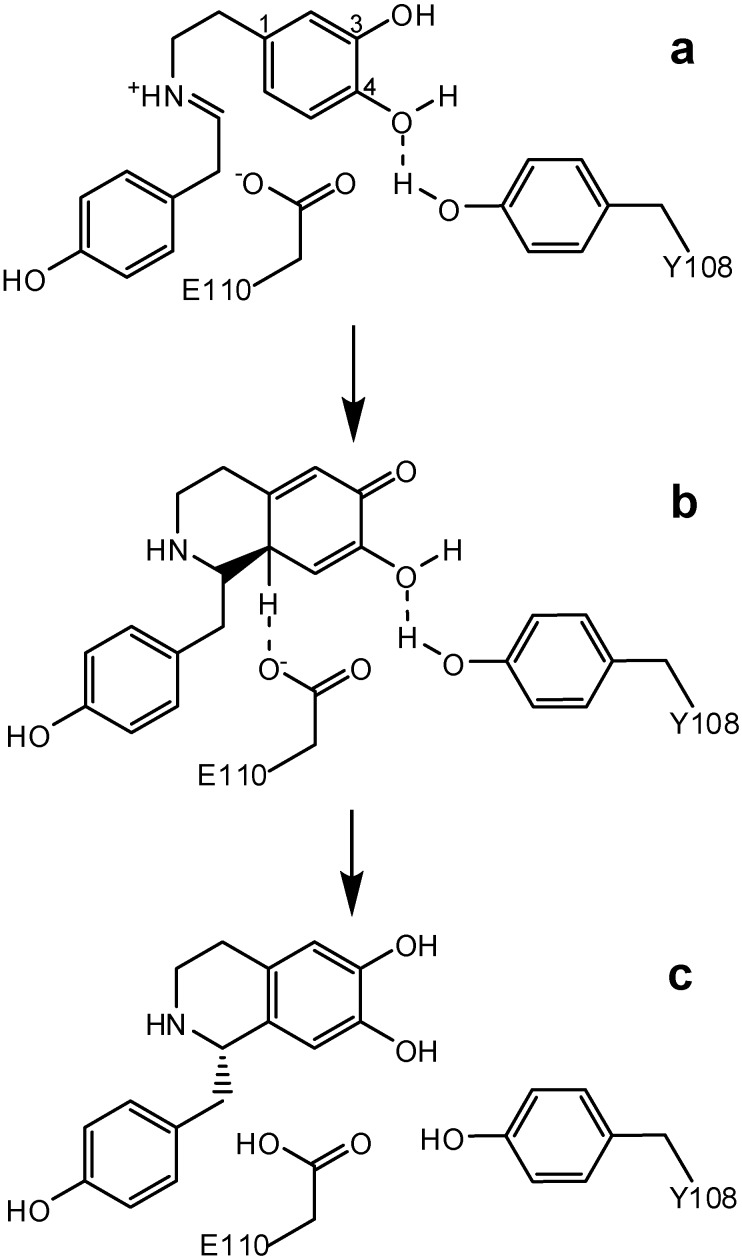
Mechanism of NCS catalyzed synthesis of (S)-norcoclaurine.

The geometry of the active site dictates a rotameric rearrangement of the iminium ion, bringing it in proximity of C2 atom of catecholate moiety and promoting the Pictet-Spengler reaction (Scheme 3, step a). The last step of NCS bifunctional catalysis is assisted by Glu^110^. This residue acts as a proton acceptor, being able to abstract hydrogen from C2, leading to the *S*-stereospecific product (steps b and c). 

This reaction mechanism is in agreement with the reaction scheme proposed by Luk *et al.* [15], based on kinetic isotope effects. The authors suggested that C2 deprotonation is driven by the transient formation of a phenolate ion (C3 phenolate) that attacks the iminium ion in the first step of the aromatic substitution process (see Scheme 3). Solvent water molecules from the wider opening of the catalytic tunnel eventually scavenge the phenolic C3 proton. Thus, the cyclization step could be considered as a concerted process in which Glu^110^ acts as a base on the catecholate moiety transiently stabilized by the Tyr^108^ hydrogen bonding on the C4 hydroxyl. The mechanism here proposed is exquisitely stereospecific in that, given the position of Glu^110^ with respect to the dopamine ring orientation, C2 proton abstraction may only occur from a single possible configuration of the intermediate. 

Both kinetic and crystallographic data appear to yield a coherent two steps mechanism in which the first step (formation of the iminium ion) can be envisaged as a subsequent bifunctional catalytic process in that the reactive aldehyde is activated first by the interaction with the Lys^122^ followed by the nucleophilic attack of dopamine. In turn, the second step (cyclization and C2 deprotonation), is envisaged as a concerted bifunctional catalytic process in which C2 proton abstraction occur simultaneously with the benzylisoquinoline ring formation.

So far, in order to obtain the biologically active (*S*)-isomer diverse synthetic strategies have been employed. Such strategies are based on asymmetric catalytic approaches involving enantioselective hydrogenation of the corresponding dihydroisoquinoline intermediates by appropriate chiral metal catalysts [16]. These synthetic routes are efficient and guarantee a good enantioselectivity. Nevertheless, large scale preparations entail extensive use of organic solvents and metal catalysts that involve the need of solvent and metal recycling. Enzymatic synthesis may offer the advantage of a clean and green synthetic process in the absence of organic solvents and metal catalysts, provided that the efficiency of the process is at least comparable to that of the traditional one. NCS catalysis here described has synthetic advantages allowing lab scale (*S*)-norcoclaurine preparation. 4-HPAA can be conveniently generated by oxidative decarboxylation of tyrosine in acqueous solutions in the presence of an equimolar amount of hypochlorite. The reaction is carried out in the same buffer needed for NCS catalysis, thus suggesting that the whole (*S*)-norcoclaurine synthesis could be optimized as a one pot reaction by quickly adding dopamine and NCS to the solution containing the newly synthesized aldehyde.

As a final comment, it should be considered that the interest in (*S*)-norcoclaurine product is paramount due to its well established sympathomimetic activity and its safe pharmacological profile [17,18,19,20]. Its activity comprises positive inotropic and chronotropic effects through β-adrenoceptor stimolation and an α-adrenoceptor antagonist activity on vascular smooth muscle and blood cells These effects can be useful in treating congestive heart failure by reducing cardiac afterload, increasing inotropic potential and blood fluidity. Therefore, (S)-norcoclaurine can advantageously compete with racemic dobutamine [21,22], the current “best in class” among sympathomimetic drugs.

## 4. Conclusions

In conclusion, the reaction mechanism here described provides an example of bifunctional catalysis applied to a chemo-enzymatic synthetic strategy. This approach represents a novel, efficient tool for manufacturing plant-derived metabolites, particularly benzylisoquinoline alkaloids. Further studies are needed in order to scale up the entire process by implementing enzyme production via a fermentation step. Moreover, enzymatic engineering can broaden the substrate specificity of norcoclaurine synthase allowing the production of novel benzylisoquinoline alkaloids.
